# High-Fidelity Agent-Based Modeling to Support Prevention Decision-Making: an Open Science Approach

**DOI:** 10.1007/s11121-021-01319-3

**Published:** 2021-11-15

**Authors:** Wouter H. Vermeer, Justin D. Smith, Uri Wilensky, C. Hendricks Brown

**Affiliations:** 1 Center for Prevention Implementation Methodology for Drug Abuse and HIV, Department of Psychiatry and Behavioral Sciences, Feinberg School of Medicine, Northwestern University, Rubloff Building, Room 10-136, 750 N Lake Shore Drive, Chicago, IL 60611 USA; 2 Center for Connected Learning and Computer-Based Modeling, School of Education and Social Policy and McCormick School of Engineering, Northwestern University, Evanston, IL USA; 3 Northwestern Institute of Complex Systems, Evanston, IL USA; 4 Department of Population Health Sciences, University of Utah School of Medicine, Salt Lake City, Utah USA

**Keywords:** Agent-based models, Replication, Reliability, High-fidelity models

## Abstract

Preventing adverse health outcomes is complex due to the multi-level contexts and social systems in which these phenomena occur. To capture both the systemic effects, local determinants, and individual-level risks and protective factors simultaneously, the prevention field has called for adoption of system science methods in general and agent-based models (ABMs) specifically. While these models can provide unique and timely insight into the potential of prevention strategies, an ABM’s ability to do so depends strongly on its accuracy in capturing the phenomenon. Furthermore, for ABMs to be useful, they need to be accepted by and available to decision-makers and other stakeholders. These two attributes of accuracy and acceptability are key components of open science. To ensure the creation of high-fidelity models and reliability in their outcomes and consequent model-based decision-making, we present a set of recommendations for adopting and using this novel method. We recommend ways to include stakeholders throughout the modeling process, as well as ways to conduct model verification, validation, and replication. Examples from HIV and overdose prevention work illustrate how these recommendations can be applied.

## Introduction

Prevention research has made many advances in the last two decades, following a path taken by medical science beginning in the 1960s in building an empirical knowledge with rigorous testing of well-defined preventive interventions against standard conditions or against other competing interventions (Hill, 1961). The conduct of these rigorous experiments, conducted as efficacy or effectiveness trials, has been well established (Brown et al., 2009). Implementation research and practice build on these earlier phases of the traditional translational pipeline (Brown et al., 2017), by taking into account unique features of the delivery system and target population (Aarons et al., 2017). Communities and organizations involved in large-scale implementation research can benefit greatly from the knowledge gained regarding how best to deliver evidence-based preventive interventions in their communities. However, until evaluation of implementation strategies becomes more widespread with implementation and hybrid trials (Brown et al., 2017), their long duration and generally limited number of tested contexts negate their value to policy makers who need to make informed decisions in a timely manner to prevent impending adverse health outcomes. Perhaps, nowhere is this limitation more apparent than the national 10-year timescale for Ending the HIV Epidemic (EHE). This requires local decision-makers to reduce new infections with long-term strategies that achieve the right balance between direct protection of those without infection and preventing transmission from those already infected (i.e., Treatment as Prevention). Decision-makers cannot wait to act until rigorous implementation trials are complete. This is one place where complex system simulation models that include the etiology of disease, the evidence-based interventions, the implementation strategies, and local data can provide the knowledge required for optimal actions.

The call for complex systems simulation modeling, and more generally systems science and engineering methods (Carey et al., 2015; Czaja et al., 2016; Lich et al., 2012; Mabry & Kaplan, 2013; Valente et al., 2015; Wang et al., 2016), to better understand and address these complexities in the development and implementation of prevention programs, has been extensive. Yet, uptake specifically of agent-based modeling (ABM), a core system science methodology that is particularly useful for informing system-level prevention decision-making, is lagging as evidenced by few examples in recent years.

ABMs are computational simulation models that capture the behavior and interactions of individuals; among one another, with their local social, physical, and/or virtual environments, and within their larger social systems (Wilensky & Rand, 2015). They are useful for understanding how system trends emerge from the underlying characteristics, behaviors and interactions of individuals, the environment, and the systems in which they are embedded. ABMs are designed to incorporate heterogeneity of individuals, which allows individual risk and protective factors to be studied. What is more, by modeling the embeddedness of these individuals in their social context and social systems, context-specific risk and protective factors can be included into these models in a natural way. The capacity for ABM simulations to run with varied types of interventions and different implementation strategies allows for prediction of their impact against appropriate counterfactuals, producing contrasts between the effects of factors that are often too difficult and too expensive to obtain empirically. Simulations can also inform how to remove health disparities by comparing impact on different populations who experience various levels of risks, geographic or network positions, or environmental exposures. For example, the effectiveness of a universal classroom-based intervention to prevent conduct disorder appears to have a stronger impact when there is a higher prevalence of aggressive/disruptive behavior (Rubow et al., 2018), so ABM simulations could inform schools about the program’s likely level of benefit relative to its cost. The computational nature of ABMs allows them to capture the dynamics in a system well into the future; thus, these models can provide a predictive lens that is particularly useful to explore the future impact of preventive strategies in time horizons that are often impractical in applied research. These characteristics allow ABMs to describe emergent behavior under various systemic perturbations, making the practice of ABM a timely and resource-efficient tool to explore the effects of potential preventive interventions and the implementation strategies needed to support their delivery.

There are two general types of ABM: theory-driven and high-fidelity models. When limited local data are available, ABMs can be used for “theory-driven modeling” (Wilensky & Rand, 2015), which aims to capture the drivers of behavior within a complex system and identify potential risk groups and levers of change that steer a system toward a desirable outcome state. Identification of such levers in itself can be valuable for decision support and to rule in or out certain implementation options.

Despite the important insights gained from theory-generating models, such knowledge may be insufficient to inform how *best* to implement evidence-based prevention programs within a local context. Effective strategies encapsulate the local context, local networks and dynamics that are known to affect the implementation process, and individual-level risk and protective factors, which can only be captured by integrating local data into the model. Thus, high-fidelity models are particularly suitable for forecasting an implementation strategy’s impact at a local level. For example, we know that continued use of pre-exposure prophylaxis (PrEP), a medication to prevent contraction of HIV for those at high risk, is highly effective in preventing HIV spread through sexual transmission among men who have sex with men (McCormack et al., 2016). Yet, fully understanding the impact of PrEP interventions requires knowledge of local characteristics, including community viral load, extent of disparities, and behavior within sexual networks. High-fidelity models embrace the complexity in the system, capture critical contextual factors affecting proximal and distal prevention outcomes, and are aligned with the local context by integrating field data (Vermeer et al., 2020).

While high-fidelity simulations from ABMs can help inform decisions, it will only be useful if it provides accurate projections and is acceptable to decision-makers. These are core components of open science. Open science, defined in this paper as “… transparent and accessible knowledge that is shared and developed through collaborative networks (Vicente-Saez & Martinez-Fuentes, 2018),” provides both a vision and a mechanism for producing not only accurate models but also ones that are accepted, trusted, owned, and used by policy makers, community coalitions, and organizations to make informed decisions. As open science often addresses the interplay between humans and systems for organizing and presenting information, it is highly relevant for model-based decision support. They both require rigor and transparency. Not only will the models need to accurately represent the phenomenon being studied, the process by which these models are created, documented, and shared should facilitate replication and provide a foundation on which the field as a whole can grow. The call for increased adoption of systems science, and ABM in particular, needs recommendations describing the practices for doing so in a rigorous manner, which we provide below.

ABMs have unique ways of generating scientific evidence that differ from those used in standard statistical modeling of empirical data. In particular, “agents” are instantiated, assigned attributes, and interact with others and their environments according to rules, so one cannot directly examine the goodness-of-fit across individuals in the way longitudinal datasets offer. Consequently, there are unique aspects of open science that are appropriate for high-fidelity ABMs. ABMs have their own ways of addressing validity, reliability of outcomes, replicability, sharing of source code, and standards of reporting (Collins et al., 2015; Grimm et al., 2006; Wilensky & Rand, 2007).

Based on our shared experiences in developing the most widely used ABM platform (Wilensky, 1999), and over a decade of contributions to the push for reliable ABMs (e.g., Vermeer et al., 2020; Wilensky & Rand, 2007), we identified three major themes that we use to structure the recommendations presented in this manuscript: (1) ensuring model validity, (2) facilitating replication, and (3) acceptance, adoption, ownership, and use by stakeholders. The first two of these address how models can be understood by model builders and scientific reviewers, while the last focuses primarily on stakeholders who have deep understanding of the local contexts but not necessarily the modeling methodology.

## Recommendations for Ensuring Model Validity

We define validity of an ABM as the extent to which a model behaves in accordance to observed and intended dynamics. For ABMs, and computational models in general, checking validity has two primary components: model verification and model validation (Rand & Rust, 2011; Wilensky & Rand, 2007). Model verification involves checking whether the translation into code is in line with the conceptual model (i.e., Does the model do what it intends to do?). Model validation consists of checking whether model behaviors are realistic and align with the observed phenomenon in the real world. While model verification should naturally occur for all ABMs to prevent erroneous behavior in the code, high-fidelity models used in prevention place a premium on capturing realistic dynamics; as such, a particularly strong emphasis on model validation is warranted for these models.

### Validate Both on the Agent and System Level

ABMs are fundamentally built by describing behaviors of the individuals as they interact with one another and their environments. By design, they will cross multiple levels as system-level behaviors emerge without being formally specified in the model, which not only makes ABMs a natural fit to consider the social systems in which we live, but also provides the opportunity to validate the model at multiple levels.

Validation of a model occurs when the model matches the phenomenon the model aims to capture. Multiple standards for what is considered an adequate match can be chosen (Wilensky & Rand, 2007), but alignment needs to occur at the level of emergent (e.g., HIV incidence rate) or system-level behavior (e.g., the proportion of the population receiving rapid HIV care among those testing positive). Beyond this traditional system-level validation, high-fidelity ABMs can also validate the behavior of individuals that give rise to such dynamics. By basing individual behaviors on local field data (e.g., sexual networks), and comparing the modeled individual-level dynamics with observed ones, one can examine the overall accuracy by which the individual-level dynamics are modeled as well as the variance in such behaviors (Wilensky & Rand, 2015).

Whenever possible, we recommend high-fidelity ABMs to leverage validation using a two-step process. In the first stage, the model should be built using validated individual-level behaviors based on observed heterogeneity in individual-level field data (e.g., demographic characteristics of partners, condom use) to ensure the validity of individual-level mechanisms. In the second stage, one should test if these individual-level dynamics in fact yield the emergence of realistic system-level dynamics and explain the phenomenon observed.

We note that the feasibility of validating ABMs on the individual level is strongly conditional upon availability of local data. As such, the capacity to do so will expand with increased sharing of data between research and policy maker partners, thereby making science more transparent and open. While individual-level data to date might not be readily available in all contexts, and might be too costly to collect for modeling studies, leveraging existing research data is a valuable alternative. When individual-level data are unavailable, one may need to draw from a reasonable distribution, for which the impact can be tested in sensitivity analysis.

We describe the process of modeling a sexual network module in a Chicago-focused HIV prevention model as an example of how multi-level validation could be leveraged in practice. In this model, the primary mode of HIV transmission is sexual interactions; as such, this model relies on a network of sexual encounters where infections can occur. In our modeling, we begin with an individual level, which closely follows individual’s decision-making for seeking partners. In this approach, we used individual-level partnership formation data from a large, longitudinal study of Chicago men-who-have-sex-with-men (MSM) sexual encounters (Mustanski et al., 2019). Individual histories of partner formation and dissolution were used to generate agent behaviors and partner selection over time (i.e., matching real world data). Oftentimes, such networks are modeled by taking global network-level characteristics, such as the degree distribution (the distribution of number of ties) or the assortativity rate (the extent to which individuals partner with individuals like themselves) and translating these into distributions of rates of partnership formation and partner choice (Jenness et al., 2018). While this alternative approach aims to generate networks that match system-level characteristic of the networks (e.g., assortativity rate), it potentially misses structural details in the network that are critical for spreading dynamics (Vermeer et al., 2016). Our recommendation is to generate individual agent-level data as accurately as possible and check validity by comparing the simulated distribution of emergent properties and system behavior against observed values (e.g., confirm that the 95% confidence interval of simulated emergent behavior includes the observed value). We can test a range of system-level measures such as degree distribution and assortative rate to validate a model.

### Integrate Local Data to Increase Model Fidelity

Similar to integrating validated individual mechanisms, inclusion of local contextual factors is key to ensuring the ABM is sufficiently high fidelity for the context it studies. Parallel to the community-based developmental epidemiology approach that has long been used in prevention science (Brown & Liao, 1999; Kellam et al., 1991), we argue that modelers should use local data whenever possible and practical to represent variations in person, place, and time that occur within a locale. For example, input of demographics, residence, and mobility of agents should closely match that of the community and its geography. For example, we used as one input the racial distributions in each of Chicago’s 77 neighborhoods. While embedding local data makes models more context specific, the increased fidelity that is derived from doing so generally outweighs the loss of generalizability, especially for models that aim to support local decision-making. For such decisions, the importance of understanding the local dynamics and (potential) barriers to implementation will be critical, suggesting the use individual data alone will not suffice, and such data need to also be specific to the context that is being modeled.

When developing an ABM, it is possible that we discover inaccuracies. The previously mentioned network module based on a Chicago-based cohort study is one example, where we found that inclusion of only individual-level risk factors was insufficient to account for the observed disparities in HIV incidence and prevalence for African Americans and Latinos compared to non-Latino whites. To capture disparities more accurately, we included a module that accounted for the racially segregated neighborhood (and by extension, social) structure of Chicago, and community-level viral load, defined as the proportion of people in that neighborhood with HIV multiplied by the proportion who were not virally suppressed. By integrating these local dynamics and data, the model not only becomes more realistic, it also becomes more tailored to the local setting. As such, integration of local data into a model increases the model fidelity and the actionability of its outcomes for prevention. While the model itself become more specific via inclusion of local data, we note that the method of building and tailoring models to their context remains generalizable. As such, building these models and reporting on the process of doing so should be seen as methodological contributions to prevention and modeling sciences that can be scaled up or out with local data from other systems and locales. To facilitate these adaptations, we recommend that both the context-related and individual-level input components be included but distinct from the core model code. By this, we mean that parameter values in the code are represented by an expression and that this expression is defined (based on field data) in a separate input module. In separating input data and code, only the module responsible for translating data into expressions will need to be adapted when local data from a different context is used, while the functional model code remains intact.

### Report the Model Validation Process

For high-fidelity models in particular, their strong reliance on field data ensures that model validation is a natural part of the model-building process. Although model validation might be the norm during model building, few reports of these efforts are documented or shared with peers. By underreporting the process of model building, much of the prior work of evaluating the validity of the model (and its results) is discarded during the publication process. This exclusion obscures much of the logic employed during model building and the assumptions that underlie the model. These pieces of information are critical when attempting to replicate, modify, or build upon the model. To improve the scientific method of modeling to support prevention, we recommend reporting not only the final model and its outcomes, but also the major steps in the process of model building itself, including the efforts undertaken to validate the behavior of the modules within a model and the system-level behaviors. The recently developed TRAnsparent and Comprehensive Ecological modeling documentation guidelines, or in short TRACE (Grimm et al., 2014), support the reporting of the model-building processes by presenting model builders a structure and language to adopt in their documentation of activities undertaken as part of the building process. TRACE asks modelers to keep a notebook of their model-building efforts and organize it using 8 main elements: Problem formulation, Model description, Data evaluation, Conceptual model evaluation, Implementation verification, Model output verification, Model analysis and application, and Model output corroboration (Ayllón et al., 2021; Grimm et al., 2014). This structure can then be leveraged to present an overview of efforts. By leveraging recent developments in the capability of dynamic documents, these notebooks can be made interactive, and a true blend of code snippets, comments, and results, which can be staggered with various levels of details to fit the need of both modeling experts as well as relative novices. These capabilities increase the shareability and readability of such documents to such an extent that they can be used for the evaluation of rigor and validity.

While tracking model development using TRACE adds to the burden of model documentation during the model-building process, the upfront time commitment for tracking and tagging is paid back with dividends during the process of generating model documentation for publication. Moreover, the resulting documentation and shared model validation process increase the validity of the model itself, the rigor in the modeling process, and the transparency of the method used. TRACE documentation is geared toward ongoing modification and adaptation of models, openly presenting the modeling artifacts as building blocks for an ongoing scientific process, and facilitating replication in the process. For this reason, we highly recommend the use of tools such as TRACE. Effective use of TRACE requires it to be adopted from the onset of model development and, as such, reported use to date is low. While still in its infancy in terms of adoption, given the benefits described above, we expect tools like TRACE to become an established standard in the reporting the model development and model validation process in the coming years. Especially in prevention science, which puts a premium on validated high-fidelity models, the adoption of TRACE as a standard will help ensure the rigor of the models used and the trust in using modeling results for prevention decision-making.

### Report Misalignments and Model Shortcomings

As George Box famously said: “all models are wrong, but some are more useful than others.” While our efforts in building the Chicago HIV model have revealed that accurately integrating local individual-level data can yield extremely accurate high-fidelity models, models by their very nature abstract some of the complexity. Consequently, it is likely that some discrepancies will occur between modeled and observed behaviors. While reporting the validation process using TRACE presents an opportunity to identify such misalignments, there is no explicit section that ensures this is in fact done. Combined with an external pressure to disseminate appealing modeling outcomes, one runs the risk of misalignment being buried rather than being used as ongoing lessons. As such, we recommend devoting a section in the Model Analysis and Model Output Corroboration sections of TRACE that specifically highlight misalignment, their hypothesized causes, and potential ways they could be improved. Specifically acknowledging early misalignments and appropriate solutions ensures a critical look at the model and helps identify missed critical dimensions or variables, which may prove critical for both for decision-makers and for model building.

Early in our Chicago EHE model building, for example, we had accurate projections of 1-year incident cases, but inaccuracies in classifying the incident cases by race/ethnicity. While including community viral load and community poverty improved the models’ outcomes of incidence rate by race/ethnicity, our model only partially captured the disparities observed in practice. On one hand, this observation supports statements that traditional risk and protective factors (which are included in the model) do not explain the observed disparities; on the other hand it highlights the need for our model to improve on integrating social determinants of health. Consequently, the current version of the model foregoes making any claims about disparities and explicitly states this specific shortcoming in the model descriptions. Currently, our development aims to better understand and integrate social determinants of health to support this model to be used to make claims about addressing disparities, as it was originally intended.

### Use Model Fitting with Caution

A common modeling practice is to obscure or “address” discrepancies in alignment by introducing one or more fitting terms—what is pejoratively described in computer science as a “kluge factor.” The purpose of including such terms is to ensure the model generates results that are aligned with observed outcomes. Generally, the arguments for including such fitting terms are uncertainties in the input data, incorrect modeling assumptions, or exclusion of important variables. While there are cases in which these arguments are warranted and model fitting can be a legitimate strategy for model improvement, for ABM in general, and high-fidelity models specifically, such a strategy is generally undesirable because fitting a model’s outcomes goes against the notion of validation on multiple levels. While model results can sometimes seem more appealing, the process of fitting can obscure details needed to objectively use models for decision support and prevention.

In summarizing this section, we recommend that model documentation should include a clear distinction between ways that the model uses input data. One classification would distinguish situations in which the individual-level data are directly entered (e.g., each agent inherits one individual’s longitudinal state data over time), parameters are derived to represent the distribution of individual-level data (e.g., modeling the distribution of times to formation of new sexual relationship), parameters are copied from previous studies (e.g., rate of HIV infection per sexual encounter), and input parameters are optimized to validate an aspect of the model (e.g., kluge factors that are added to improve predictions).

### Present Outcome Variation

High-fidelity ABMs are generally stochastic, meaning they incorporate some form of randomness in the behaviors within the model. Such randomness can stem from draws from a distribution, or a random order in which individuals are activated. As a result, there can be path dependence and uncertainty about model outcomes. When uncertainty is combined with feedback loops, non-linear dynamics, and complex interactions within these models, it has the potential to cause substantial variations in outcomes across model runs. The norm for addressing variance in the outcomes is to use multiple repetitions of a given set of parameters and to report the average results across them. While considering the mean outcome is an effective way of summarizing the outcomes over multiple repetitions, it is incorrect to assume that this mean behavior is representative or even likely to occur in the system being modeled or the real-world phenomena on which it is based. The fact is that variation in the outcomes generates a range of behaviors that can occur, and ignoring such variance by presenting only mean behaviors provides a false sense of the stability of these behaviors. Awareness of the fact that mean behavior might not be the most representative, or even useful, outcome is critical for interpreting model results, especially for decision support.

In reporting model behaviors, we explicitly call for providing relevant quantiles (e.g., median, maximum, and minimum) and other summary statistics in TRACE’s Model Analysis section. For example, consider checking whether the distribution of R simulated incident counts *Y*_1_, …, *Y*_R_ is in agreement with the observed number of incident cases 1 year later, *N*. A straightforward comparison would be to form a 95% confidence interval, for the difference, i.e., $(\bar{Y }-N)\pm 1.96 \sqrt{{var}(Y)/ R + N}$ and see if it contains the null value of 0. This would reveal if the mean of the simulation deviates from the observed value, taking into account variation in both the simulations and the observed value. More relevant statistical indices would examine how variable the individual simulations were. For example, we could compute the number of simulations where the individual confidence intervals $({Y}_{i}-N)\pm 1.96 \sqrt{{Y}_{i} + N}$ excluded 0. Simulations having only 5% of these intervals exclude 0 are numerically aligned to the observed data, but models with 10–15% exclusions are reasonably representative as well. Alternatively, presenting the distribution of simulated effect sizes, i.e., ratios $({Y}_{i}-N)/\sqrt{{Y}_{i} + N}$, would show the full range of simulated versus observed differences.

### Evaluate Model Robustness

We want to highlight one core piece of the TRACE specifically. The key purpose of the Model Analysis section in TRACE is to report the robustness of the modeled outcomes, which is key to understanding how to use modeled results in decision-making. Similar to the variation across runs caused by stochasticity, the model robustness refers to the extent to which model dynamics and outcomes will vary as a result of perturbations in the modules or inputs used. There are multiple reasons why uncertainty exists regarding the implementation of a mechanism or module. Either the input data used to calibrate the module is weaker or unavailable, or the exact functioning of a mechanism is not clearly documented and one interpretation was selected. Regardless of the cause, the aim is to report the impact of such uncertainties on the outcomes and in the interpretation of model results. As such, a sensitivity analysis should always be conducted in which one perturbs the less robust parameters or mechanisms in the model and analyzes the impact of such changes on model outcomes.

This sensitivity analysis should be reported as part of any dissemination of modeling results as it provides an indicator of the overall stability of model outcomes. While highly sensitive models are not necessarily problematic, it is vital to know the extent to which a model’s outcomes are conditional upon the chosen parameterization or behavior. A more cautious interpretation of results is appropriate for highly sensitive models, warranting a more critical look at the validation of its mechanisms, and potentially relying more on its relative effects rather than the effect sizes. In contrast, highly robust results can mitigate potential concerns relating to validation of the model. As a sensitivity analysis by design examines the impact of perturbations to a system, it is an invaluable tool for identifying critical levers for changing systemic behavior. Additionally, it is likely to provide bounds to the extent to which certain intervention can change systemic behavior. Both pieces of information are useful for decision-makers, but can only be leveraged if sensitivity analysis becomes a central piece of the modeling outcomes reported. Consequently, we recommend sensitivity analysis to be a key element of the modeling effort for any ABM used in the prevention science domain.

## Recommendations for Facilitating Replication

Replication has been a core practice of the scientific method for as long as research has been conducted. By replicating experiments and comparing results across independent studies, scientists have been able to verify protocols and findings and create reliable knowledge for future research to build upon. As such, replication has been a foundational principle allowing for accumulation of knowledge. For simulation models, which by their very nature create a virtual representation of the phenomenon under study, replication is a natural way of checking the accuracy of the behaviors of these models; therefore, it is a natural process that accounts for model verification and model validation. When model accuracy comes at a premium, as is the case when high-fidelity models are used for decision support and prevention, ensuring reliability of model dynamics by means of replication becomes critically important. What is more, the need for replication of such models is higher than ever before. Without replication, we cannot have confidence in model findings, thus risking the integrity of the foundation of the body of work. Adopting ABM without replication can degrade the credibility of the method as a whole and hamper the potential of it to advance prevention science. To ensure the potential of ABM in prevention can be reached, we present recommendations on how to facilitate replication below.

### Support a Culture of Replication

While the value of replication has long been recognized in modeling literature (Axtell et al., 1996; Edmonds & Hales, 2003; Thiele & Grimm, 2015; Wilensky & Rand, 2007, 2015), there is a surprisingly limited amount of replication and validation of ABMs in the literature. This limited presence can be attributed in a large part to poor attribution of value to replication studies and an incentive structure in the publication culture that primarily rewards novelty.

Currently, journals are most like to publish research when it presents novel or counterintuitive results. Replication studies, by definition being neither original nor aimed at adding new knowledge, are poorly aligned with this dominant view of what is publishable research. For replication to play a viable part in our scientific process, this perspective of what is considered valuable for science will need to change. Replication efforts will need to be both embraced in publication culture and credited as solidifying the existing knowledge base.

Checking the conclusions of each high-fidelity ABM by full replication is highly impractical owing to the extreme effort such an effort would require. Our own project of completely replicating a high-fidelity HIV model (Vermeer et al., 2020) spanned a period of 18 months, involved three team members, and required multiple communications between our team and the original model creation team, and involved sharing of models, source code, and proper documentation. Although modular replication (see next section) can reduce some of this load, it remains infeasible for replication to become a systematic part of the peer review process, especially for high-fidelity models.

While integrating replication as part of the review process is a bridge too far, there are changes that can be made to improve current publishing standards. We recommend publishers to require documentation to include TRACE (Grimm et al., 2014) and The Overview Design Details standard (ODD) (Grimm et al., 2006, 2010) for all published models. In doing so publishers can create a pull for more rigorous models, allow for better replication of these models, and hold modelers accountable for their *process*, not just the results, of modeling.

### Leverage Modularity

In our process of replicating a high-fidelity model for HIV prevention, we found that leveraging modularity can reduce the efforts required for replication. High-fidelity models are complex and capture a multitude of dimensions and contextual factors. With many dimensions come many interactions, making fully understanding dynamics and model structure difficult for high-fidelity models. Dividing the model code into smaller segments, which we refer to as modules, and replicating one module at a time reduce the complexity of the replication process. Each module’s behavior can be verified without the need to completely understand the complete model. Model builders can facilitate this process by building their models with a modular structure in mind, writing sections of code that are distinct into modules and explicitly highlighting the interaction between such modules. The adoption of functional code lends itself well for building modular structures, as by definition each function is structured to have defined inputs and outputs, allowing it to be specified as a module. An added benefit of fixing input and output structures is that each module can be altered or replaced with a updated version whenever new knowledge or data becomes available without impacting the remainder of the model, facilitating tailoring of the model in future modifications. Consequently, modular models and functional code allow for both easier integration of new knowledge, easier model validation, and better grasping the structure of a high-fidelity model, all of which can be leveraged to ease the replication process.

### Standardize Model Documentation

A sense of model hierarchy and structure is required to leverage modularity during replication. Such insight can only be conveyed efficiently by appropriate standardized model documentation. ODD (Grimm et al., 2006, 2010) is a widely adopted standard for documentation of ABMs and is rapidly gaining traction. While we strongly encourage the use of this standard, many of the details and interactions that occur in high-fidelity models are encapsulated into the “sub-models” section, which makes it fall short in providing an overview of the model structure (Grimm et al., 2020). Consequently, we recommend inclusion of a modular flow diagram in the Overview section of the ODD protocol. Such a diagram depicts the modules present in the model and connects the modules that impact one another. In doing so, it presents a graphical overview of the model structure that complements the process overview, which is generally described by means of pseudo code. Presenting such an overview not only facilitates the modularization of the model during replication, it also provides a structure that can be followed and referred back to when describing sub-models in the documentation. Displaying both a modular flow diagram and sub-model structure aids in understanding a high-fidelity model.

### Make Model Code Publicly Available

Complex (social) systems often exhibit non-linear behaviors and tipping points that are sensitive to small differences in model details. As such, high-fidelity model replications can potentially produce different results even if they are very similar to the original. Such a lack of robustness would be important to know. Exact replication across platforms and languages is known to be extremely difficult (e.g., Miodownika et al., 2010) and is further hampered by the inherent difficulties in model reporting. Given the sheer amount of description needed to capture all nuances and interactions of a high-fidelity model, even the best documentation is likely to fall short in providing the details needed for perfect replication. This makes it nearly inevitable that differences will occur during translation from text to code. The ambiguity in the translation process dictates that replication of high-fidelity models require sources of information that go beyond a plain English description of the model.

For this reason, we recommend three additional resources to be made available for replicators. First, the aforementioned documentation of the model validation process. Second, for model builders to be available for potential questions from replicators. Our replication efforts have shown that being able to tap this “insider knowledge” allows for a much more dynamic interpretation of the documentation and address questions relating to interpretation of the written word, which is extremely valuable during the translation process (Vermeer et al., 2020). And third, model code needs to be made available as part of the dissemination process. Sharing of code has two major advantages. It is the only way to guarantee what the model is doing, as the actual machine interpreted code can be viewed and compared. Moreover, the availability of model code allows one to do model verification concurrently with the replication process. While we can do validation based on observed module or model outcomes, verification requires a look at the actual code being implemented and thus can only occur when such code is shared.

Sharing code has long been the gold standard within the modeling domain. However, while generally being perceived as widely beneficial (Collins et al., 2015), it is far from the being the norm. Only in recent years have we observed a change in policy, with sharing of model code more often seen as a required element for dissemination of results—a trend we strongly encourage.

### Share Input Data when Possible

In line with creating transparency and facilitating replication by sharing model code, we also encourage the inclusion of input data as part of the material being shared. As stated by the editors of the journal *Science* (Hanson et al., 2011), “*It is obvious that making data widely available is an essential element of scientific research. The scientific community strives to meet its basic responsibilities toward transparency, standardization, and data archiving.*” However, input data often contains individual-level characteristics, has proprietary elements, or is not owned by the model builders. The inability to share data, regardless of the reason, poses a potential tension as local nuances in this data are likely to affect modeled behavior and are required to assess the validity of the model in capturing the local context and individual behaviors. Consequently, in cases where raw data cannot be made publicly available, we suggest taking advantage of advances in data curation (Borgman, 2012; Hanson et al., 2011; Kum et al., 2011; Olson et al., 2008; Palmer et al., 2007) such as engaging in a process of preparing input data in the form of summary statistics and distributions that can be shared as part of the publication process, as well as preparing and archiving data (Whitlock, 2011). We recommend including owners of the data as coauthors in dissemination, as this promotes data sharing (where input data may be requested) and engenders confidence that the data will be used appropriately.

### Community Acceptance, Adoption, Ownership, and Use of Model-Based Decision-Making

Many policy makers are familiar with and use “data driven decision-making,” a core of epidemiology that is based on efficacy/effectiveness evidence-based findings. We envision another tier that we call “model driven decision-making.” This approach relies on simulation studies and “what-if” alternatives to answer community needs that extend the existing evidence base across time and context in ways that field trials can never complete in time for timely decision-making. While the creation of high-fidelity models should go hand-in-hand with the use of models for decision support, it will be policy makers and community representatives, not the model builders, who will be making decisions to act upon the modeled results. As such, the high-fidelity modeling approach specifically, and the open science movement in general, cannot ignore the role of policy makers and community opinion leaders in its standards. We argue that for models to be used for decision support, those developing the models need to partner with community partners and other stakeholders, work under the aegis of their organizations and systems, and facilitate understanding and use of these models to make decisions. Given this goal, it will be critical to engage stakeholders early in the model-building process rather than to see them as merely the recipients of the results.

We provide an example of this approach through our modeling efforts to study the impact of COVID-19 on opioid use-related mortality in Pinellas FL. We became members of the local task force that was assembled to reduce overdose deaths. The task force included treatment providers, service system leaders, community advocates, and the county health department as partners. Some of the financial resources of this study (provided by the National Institute on Drug Abuse (NIDA)) were used to support weekly feedback from the task force’s two co-chairs, following principles of community-engaged research in prevention (Brown et al., 2012; Kellam, 2012). The benefits we observed from this practice were twofold. First, working with the local experts allowed us to identify and prioritize interventions and implementation strategies that are feasible (e.g., what programs can be funded legally by local, state, or federal government dollars). Being immersed in the field, our partners had the best sense of which modeled mechanisms were realistic or not, allowing for a rich source of model validation during the model-building process. Second, community questions were translated into model experiments, answering specific questions for which our stakeholders lacked the answer, and directly impacted their activities. Providing a direct response to the local needs facilitated community acceptance and adoption of the model during the decision-making process.

We argue that when models are built for supporting stakeholder decision-making, these stakeholders should be made a part of the model-building process. Researchers cannot afford to ignore stakeholders’ value as resources, nor their role in the dissemination to and adoption of the practices. Instead, stakeholders should be engaged throughout the modeling process to ensure the model’s practical relevance and impact.

## Conclusion

The call for increased adoption of systems science methods in general, and agent-based simulation models specifically, in prevention science can only be successful when adoption of this novel methodology is done with sufficient rigor. In this paper, we presented a set of recommendations, summarized in Table 1, to guide the field in doing just so.

**Table 1:** Overview of recommendations for rigorous high-fidelity agent-based modeling in prevention science

	**Subdomains**	**Action steps**
**1** **Ensuring model validity**	a. Validate both on the agent and system level	- Build models using agent behaviors based on individual data and validate emerging system dynamics
	b. Increase model fidelity by integrating local data	- Build models using data that is specific to the context that is studied
	c. Report the validation process	- Adopt TRACE standard to report the modeling and validation process
	d. Report misalignments and model shortcomings	- Include a specific on misalignments, in the Model Output Corroboration section of TRACE
	e. Use model fitting with caution	- Caution against including fitting terms without a good reason
	f. Present outcome variance	- Always present a distribution of results and discuss the variability of model outcomes
	g. Evaluate model robustness	- Always include a sensitivity analysis as part of disseminated modeling results
**2** **Facilitating replication**	a. Support a culture of replication	- Require ODD and TRACE documents as part of the publication process
	b. Leverage modularity	- Reduce complexity of replication by replicating one module at a time
	c. Standardize model documentation	- Adopt ODD in documentation - Include a modular flow diagram in the Overview section of the ODD
	d. Make model code publicly available	- Share as many artifacts of the modeling process as possible, including TRACE, ODD, and modeling code, and input data - Be willing to answer additional validation and replication questions
	e. Share input data when possible	
**3** **Acceptance, adoption, ownership, and use by stakeholders**	a. Include community partners in the model-building process	- Partner with community stakeholders as early as possible in the modeling process - Focus on answering locally relevant questions - Leverage local stakeholder knowledge for model validation

What becomes evident when considering this set of recommendations as a whole is that it represents an effort to be more transparent and open in what is modeled and how it is modeled. These recommendations consider one’s modeling work as part of a larger process of building a generalizable knowledge base, to extend from and build upon, and ultimately support the rigor of the method and the field as a whole. While this perspective is highly relevant for prevention science as it embarks on integrating a novel methodology into its repertoire, it should be noted that such guidance generalizes to complex systems beyond the boundaries of prevention science. Moreover, sharing models and data, presenting more detailed reports of validation processes, and setting standards for reporting (both the model and its outcomes) all facilitate replication and align with fundamental notions of open science.

While we have noted several benefits of ABM, we should emphasize it is not the silver bullet for all prevention science problems. We consider ABM to contribute to studies where the system or phenomenon studied entails at least one the following four characteristics: (1) multiple levels of behavior or organization; (2) interactions among the elements of the system; (3) environmental attributes that impact behaviors; or (4) heterogeneity influences observed behavior. Without these characteristics, adoption of ABM, while still feasible, is likely of little additional value to alternative methods.

We also note that there are many prevention settings that are ripe for, but have yet to use, ABM in any comprehensive way. For example, we are exploring how peer leader and network-based interventions can succeed or fail and the impact of what we call multiplicative interventions (Brown et al., Under review). There is little empirical knowledge of how many peer leaders are needed and where they should be situated in the school friendship network to touch the lives of those who are on the periphery where the most suicidal youth are typically located (Pickering et al., 2018). Simulation models can fill in gaps that randomized trials can never answer.

Another challenging prevention issue that could benefit from ABM is to model factors that would contribute to equity for minoritized and low-income populations (McNulty et al., 2019). Prevention science has largely focused on intervening to promote the health of individuals who experience disparities, such as sexual and gender minorities. Intervention trials that target these populations are easier to conduct than those that could take on broader social inequalities that result from structural racism, sexism, and homophobia, but these broader interventions may be necessary to shift population disparities. ABMs could contribute important knowledge about which social strategies could work to achieve equitable implementation in service of eliminating health disparities (Smith et al., In Press). Finally, we note that ABMs could provide valuable information for social strategies to increase the use of vaccines and other biomedical interventions for prevention, especially in communities of color.
